# Hair cortisol in polycystic ovary syndrome

**DOI:** 10.1038/s41598-022-14061-9

**Published:** 2022-06-20

**Authors:** D. Gonzalez, P. Maidana, C. Ibar, J. Jamardo, D. Jacobsen, A. Fritzler, F. Fortuna, G. Fernandez, E. Lamas-Majek, S. Mallea-Gil, C. Ballarino, C. Onetto, M. Lopez, Viviana Mesch, B. Fabre

**Affiliations:** 1 Departamento de Bioquímica Clínica-Instituto de Fisiopatología y Bioquímica Clínica (INFIBIOC), Facultad de Farmacia y Bioquímica, Universidad de Buenos Aires, 956 (1113) Junín, Buenos Aires Argentina; 2 División Ginecología, Hospital de Clínicas “José de San Martín”, Universidad de Buenos Aires, 2351 (1120) Córdoba, Buenos Aires Argentina; 3 Servicio de Endocrinología, Hospital Militar Central, Luis María Campos, 726 (1426) Buenos Aires, Argentina

**Keywords:** Biochemistry, Endocrinology

## Abstract

The aim of the study was to evaluate adrenal axis hyperactivation measuring hair cortisol levels, and its influence on the relationship among metabolic parameters, inflammation markers and androgens in adult women with PCOS. 44 women (18–34 years) with PCOS diagnosis and a control group of 49 healthy women (19–35 years) were included. In both gropus body mass index (BMI) was calculated and waist circumference (WC) was measured. Hair cortisol, total serum testosterone (TT), serum cortisol, 25 OH vitamin D (25OHD), insulin, high sensitivity C-reactive protein (hsCRP), triglycerides (TG), HDL cholesterol (HDL), glucose and leptin were measured. Bioavailable testosterone (bioT) was calculated. Hair cortisol concentration was higher and significantly different in PCOS patients compared to the control group (130 vs 63 pg/mg of hair, p < 0.001). Subsequently, patients with PCOS were divided into two groups according to hair cortisol levels: group 1 with normal hair cortisol concentration and group 2 with levels above the upper limit of the reference values (128 pg/mg of hair). In group 2, TT significantly correlated with 25OHD, hsCRP, TG/HDL index, BMI, WC, insulin and HOMA (p < 0.05); bioT correlated with hsCRP and leptin (p < 0.05). Finally, 25OHD was inversely correlated with leptin and with TG/HDL index (p < 0.05). High hair cortisol concentration in patients with PCOS confirmed hyperactivation of the HPA axis. The associations observed were only found in patients with PCOS with high hair cortisol levels (> 128 pg/mg of hair), showing a possible effect of HPA axis in these associations.

## Introduction

Polycystic ovary syndrome (PCOS) is the most common cause of hyperandrogenism, with an incidence of 5–10% in both adult women and adolescents. It is a heterogeneous disorder which has been recognized as the most common endocrinopathy in reproductive age women^1^. Given its complex pathophysiology, PCOS would respond to a very heterogeneous etiology, where a variety of genetic and environmental determinants would combine^2^.

The consequences of androgens excess at metabolic level in patients with PCOS are manifested with dyslipidemia, glucose intolerance and insulin resistance^3^, which increases the probability of presenting metabolic syndrome and consequently, cardiovascular disease and type 2 diabetes. These patients also tend to present a low-grade chronic inflammation that could be the link between the presence of this pathology and its long-term metabolic complications^4^. Several authors also find an increase of inflammation markers in PCOS, although the results are controversial^5,6^.

Among the metabolic alterations that women with PCOS may present, a 25 OH vitamin D (25OHD) deficiency has also been described, which could be related to the pathogenesis of insulin resistance in this disease and to metabolic risk factors^7–9^. Beyond its classic role in bone metabolism, nowadays, 25OHD actions are described in multiple tissues and it has been related to several diseases. Its receptors are ubiquitous and can be found in adipose tissue, cardiac and skeletal muscle, pancreatic cells^10^, as well as in the ovary and the male genital tract^11^, suggesting a role of vitamin D in female reproductive processes^12^. Numerous studies have also shown inverse associations between 25OHD and inflammation markers^13,14^. Since it has a modulating effect on the immune system, hypovitaminosis D could induce a greater inflammatory response^15^. However, there are very few publications in which the association between 25OHD and inflammation markers were evaluated in PCOS patients.

It is well known that exposure to excessive concentrations of androgens during fetal life in animal models produces characteristics that mimic PCOS in adults^2^. On the other hand, it has been postulated that the exposure of the fetus or neonate to the action of an excess of glucocorticoids in a critical moment of their maturation can lead to the development of certain pathologies^16^. Endogenous glucocorticoid levels may increase as a result of malnutrition or depression in the mother, and in the fetus and neonate as a consequence of stress, for example from hypoxia. Glucocorticoids excess in the fetus may also be due to the mother being treated with these hormones. The hypothalamic–pituitary–adrenal (HPA) axis has a critical role in the regulation of cardiovascular, reproductive, neurological and metabolic systems and its dysregulation is associated with chronic diseases such as diabetes mellitus, obesity and cardiovascular disease, as well as psychiatric illnesses^17,18^. Pasquali et al.^19^ have described that HPA axis hyperactivity may be present in women with PCOS as well as an alteration in cortisol pathways, even though cortisol serum levels might be normal.

Some inflammation markers such as C-reactive protein (CRP) as well as adipokines like leptin could also be involved in the development of these processes. It should also be taken into account that the impact of these patophysiological mechanisms may be different in overweight or obese women with PCOS than in those with normal weight.

In the last decade, hair cortisol measurement has been proposed as a novel biomarker for the evaluation of adrenal axis function^20–24^. Since cortisol measurement in other samples (blood, saliva, urine) only provide specific information of a particular moment or over a short period of time, studies have been developed using other matrices that make it possible to estimate retrospective cortisol systemic concentration in a longer period of time. There is numerous evidence that attributes this advantage to the hair sample. It is postulated that 3 cm of hair would reflect the cortisol levels to which the individual was exposed in the last 3 months, since hair grows approximately 1 cm per month^20,21^. It is noteworthy that no studies evaluating the relationship between the hyperactivation of HPA axis and metabolic and inflammatory alterations in women with PCOS have been developed.

The aim of this study was to evaluate if hyperactivation of adrenal axis, evidenced by hair cortisol levels, influence the relationship among metabolic parameters, inflammation markers and androgens in women with PCOS in adult life.

## Patients and methods

In order to perform this study, 44 adult women (18–34 years) diagnosed with PCOS according to Rotterdam criteria were included.

### Exclusion criteria

Pregnancy, treatment with oral contraceptives or hormonal replacement therapy, corticosteroids or any other drug that modified lipids metabolism in the previous three months, diabetes, kidney, liver or thyroid disease, hormone dependent tumors, vaginal bleeding of unknown etiology and cardiovascular disease. In no case did alcohol consumption exceed 10 g/day and they did not consume tobacco.

We also recruited a control group constituted by 49 healthy women (19–35 years), with regular menstrual cycles, with no hyperandrogenemia or hirsutism and without pharmacological treatment.

Blood samples were obtained in the early follicular phase (day 3–5 of the menstrual cycle), after 12 h of fasting and between 8 and 9 am.

The study was approved by the Ethics Committee of the Hospital de Clínicas “José de San Martín”, Universidad de Buenos Aires and was conducted in accordance with the 1964 Helsinki declaration and its later amendments or comparable ethical standards. All patients gave their written informed consent to participate.

### Anthropometric parameters

Body mass index (BMI) was calculated in all patients and waist circumference (WC) was measured as an indicator of abdominal obesity.

### Biochemical parameters

Total testosterone (TT) was determined by electrochemiluminescence (Cobas e-411), CVs intra and interasssay lower than 4.7% and 8.4% respectively; bioavailable testosterone (bioT) and free testosterone (fT) by calculation from TT and SHBG using the Vermeulen equation^25^. SHBG was assessed by a chemiluminescent method (Immulite 2000), CVs intra and interasssay lower than 5.3% and 6.6%, respectively; serum cortisol via chemiluminescent method (Immulite 2000), CVs intra and interasssay lower than 7.4% and 9.4%, respectively; high sensitivity CRP (hsCRP) by immunoturbidimetry on a Cobas 6000 autoanalyzer, CVs intra and interasssay lower than 1.3% and 5.7%, respectively; leptin by ELISA DiaSource, CVs intra and interasssay lower than 13.3% and 12.7%, respectively; 25OHD by a chemiluminescent method (Advia Centaur^®^ XPT), CVs intra and interasssay lower than 4.7% and 11.9% respectively and insulin by a chemiluminescent method (Architect i 1000), CVs intra and interasssay lower than 4.2% and 5.2%, respectively. The degree of insulin resistance was determined through the TG (tryglicerides)/ HDL cholesterol (HDL) index and HOMA index (insulin (μIU/L) × glucose (mmol/L)/22.5). Regarding TG/HDL index, in 2003 Mc. Loughlin et al.^26^ proposed it as a surrogate marker of insulin resistance in overweight individuals, highlighting the usefulness of these two easily measured variables. They proposed a cut-point of 1.8, with showed respective sensitivity and specifity of 64% and 68%.

TG, HDL and glucose were measured by enzymatic colorimetric methods (Cobas C501). CVs intra and interasssay were 1.2% and 2.0% (TG), 1.0% and 1.5% (HDL), and 1.0% and 1.3% (glucose), respectively. The lipid accumulation product (LAP): [waist (cm) − 58] × triglycerides (mmol/L) was calculated as an indicator of insulin resistance and metabolic risk. Hair samples were obtained with scissors from the posterior vertex as close to the scalp as possible. Considering that hair grows approximately 1 cm per month, 3 cm were obtained in order to evaluate hair cortisol levels of the last 3 months. Each sample was stored in a paper envelope at room temperature until it was processed. Once the samples were obtained, three centimeters were measured from the root segment adjacent to the cutting. Then, each sample was weighed, and cortisol was extracted and processed by an automated chemiluminescent method (Immulite 2000 autoanalyzer, Siemens, LA, USA) according to the procedure developed in our laboratory. The results were expressed in pg/mg. Hair cortisol concentration reference interval in healthy individuals with low levels of stress is 40–128 pg/mg hair (P2.5–P97.5)^21^. CVs intra and interasssay lower than 15% and 20%, respectively^21^.

### Statistical analysis

For evaluating if the variables had a parametric distribution, Kolmogorov–Smirnov normality test was used. The correlation between the different variables was calculated using the Pearson or Spearman test, according to their distribution. Mann Whitney test was applied to evaluate differences between medians. A p < 0.05 was considered statistically significant. Statistical analysis was performed using SPSS 23 software (IBM).

## Results

Table 1 shows antropometric and biochemical characteristics of the two studied groups.

**Table 1:** Results are expressed as median (range) or mean ± SD according to distribution.

	PCOS	Control	p
Age (years)	29 ± 5	26 ± 4	0.075
Weight (kg)	76 ± 19	51 ± 5	< 0.001
Height (m)	1.59 ± 0.6	1.59 ± 0.7	0.851
BMI (kg/m^2^)	29.6 ± 7.0	20.3 ± 1.6	< 0.001
WC (cm)	94.1 ± 15.9	68.3 ± 10.9	0.002
Glu (mmol/L)	5.08 ± 0.56	5.06 ± 0.53	0.943
Insulin (pmol/L)	96.76 ± 84.44	44.03 ± 14.23	0.179
HOMA	3.21 ± 3.36	1.48 ± 0.52	0.311
TG (mmol/L)	1.30 ± 0.69	0.90 ± 0.39	0.194
HDL (mmol/L)	1.25 ± 0.33	1.55 ± 0.27	0.005
TG/HDL	2.7 ± 1.9	1.2 ± 0.4	< 0.001
hs-CRP (nmol/L)	33.62 ± 33.97	11.71 ± 7.61	< 0.001
LAP (cm mmol/L)	55.8 ± 42.5	12.6 ± 0.5	< 0.001
TT (nmol/L)	1.42 ± 0.34	0.94 ± 0.60	0.001
fT (pmol/L)	28.7 ± 17.09	11.16 ± 7.52	0.007
bioT (nmol/L)	0.65 ± 0.39	0.26 ± 0.17	0.008
SHBG (nmol/L)	36.28 ± 25.63	61.76 ± 32.16	0.006
25OHD (nmol/L)	37.5 (25–97.5)	43.4 (25–97)	0.017
Leptin (ng/mL)	29.5 ± 15.8	17.3 ± 13.6	0.059
Cortisol 8hs (nmol/L)	344.8 ± 111.4	366.9 ± 139.3	0.618
Hair cortisol (pg/mg)	130 (40–1106)	63 (40–128)	< 0.001

*BMI* body mass index, *WC* waist circumference, *Glu* glucose, *TG* tryglicerides, *HDL* HDL cholesterol, *hs-CRP* high sensitive C reactive protein, *LAP* lipid accumulation product, *TT* total testosterone, *fT* free testosterone, *bioT* bioavailable testosterone, *25OHD* 25-hydroxyvitamin D.

**Figure 1:**
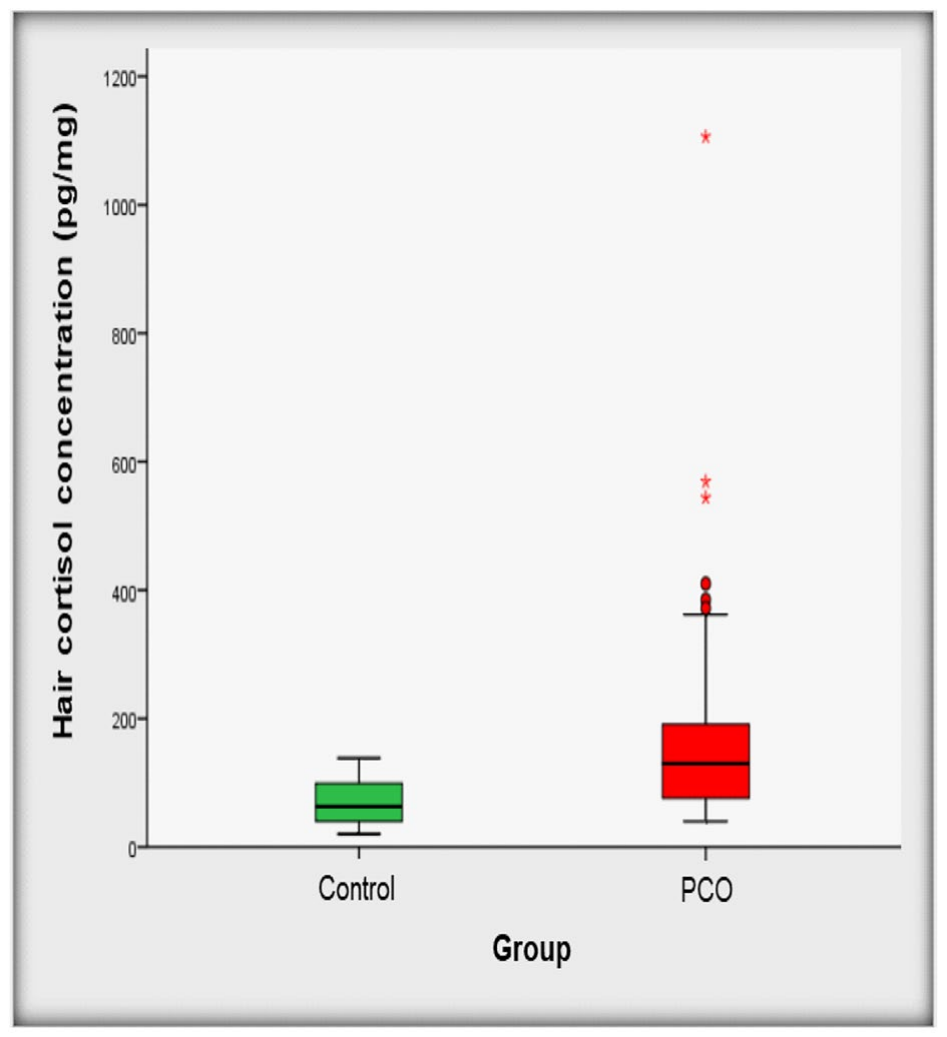
Hair cortisol concentration in the studied population.

Serum cortisol in PCOS patients was 344.8 ± 111.4 nmol/L, being within the reference values ​​(138.0–689.8 nmol/L). Hair cortisol concentration in this group was higher and significantly different compared to the control group (130 vs 63 pg/mg of hair, p < 0.001) Fig. 1.

**Figure 2:**
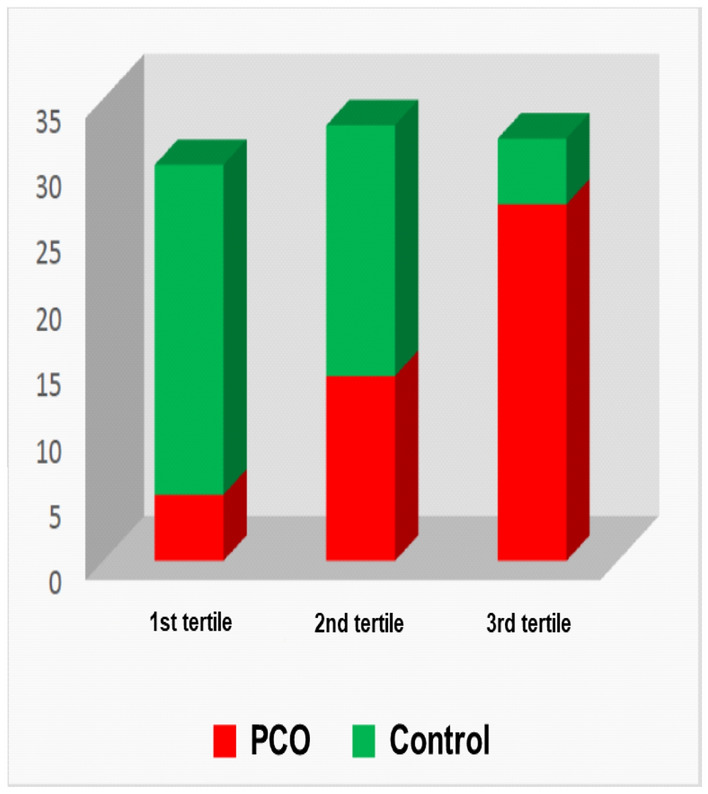
Distribution of patients and controls according to the tertiles of hair cortisol.

When the population was divided into tertiles according to hair cortisol concentration, it was found that women with PCOS were more frequently in the 3rd tertile of hair cortisol concentration (values ​​greater than 117 pg/mg of hair) Fig. 2.

Associations were found between BMI and LAP (r = 0.81, p < 0.001), HOMA (r = 0.74, p < 0.001), TG/HDL (r = 0.63, p < 0.001), TT (r = 0.49, p < 0.001, fT (p = 0.70, p < 0.001) and bioT (r = 0.70, p < 0.001).

No associations between either serum or hair cortisol with the variables studied were found. Subsequently, patients with PCOS were divided into two groups according to hair cortisol levels: group 1 (n: 21), with normal hair cortisol and group 2 (n: 23) with levels above the upper limit of the reference value (128 pg/mg of hair)^20^. In group 2 it was found that To correlated significantly with: 25OHD (r = − 0.315, p = 0.04), hsCRP (r = 0.526, p = 0.006), TG/HDL index (r = 0.328, p = 0.034), BMI (r = 0.545, p < 0.001), WC (r = 0.465, p = 0.04), insulin (r = 0.547, p = 0.001), HOMA index (r = 0.495, p = 0.005) and LAP (r = 0.457, p = 0.009). BioT showed significant correlation with hsCRP and leptin (r = 0.433, p = 0.03; r = 0.514, p = 0.014, respectively). Finally, 25OHD was inversely correlated with leptin and with TG/HDL index (r = − 0.408, p = 0.048; r = − 0.417, p = 0.004, respectively). These correlations were not found in group 1.

## Discussion

Investigating HPA axis chronic hyperactivity in PCOS is difficult due to the lack of an adequate biomarker. Since cortisol is the hormone of choice in the evaluation of the HPA axis, it should be taken into account that circulating concentrations are highly variable due to its pulsatile secretion and circadian rhythm^27^. With regard to serum cortisol, its levels in PCOS patients were within the reference range, as well as no associations with the variables studied were found. However, we found increased hair cortisol values ​​in the PCOS group when compared to the control group. Adrenal axis hyperactivation in adult life could be due to a programming of this axis in early stages of life caused by exposure to high concentrations of glucocorticoids, endogenous or even exogenous, as a consequence of maternal stress during pregnancy. This situation would determine an increased risk of developing an hyperandrogenic phenotype, as occurs in PCOS resulting in a fundamental link between very early stages of life and the development of long-term chronic diseases^28^. Epigenetics is postulated as the mechanism by which this phenomenon would occur, through DNA methylation and/or histone acetylation processes, which would lead to the modification of the expression of genes related to HPA axis regulation^29^.

Several investigations show an association of PCOS with unfavorable metabolic and inflammatory profiles. The excess of androgens characteristic of this pathology correlates with an increase in abdominal fat deposition^30^, which is also associated with hyperinsulinemia and insulin resistance, perpetuating this unfavorable condition for women's health. Currently, PCOS is considered a state of chronic inflammation that is characterized by increased circulation of molecules such as tumor necrosis factor-α (TNF-α), interleukin (IL)-6, IL-1 and CRP among others, which act directly or indirectly as mediators of systemic inflammation. In a previous study we had already found lower vitamin D levels in PCOS patients than in control group and vitamin D inversely correlated with TT, bioT and with LAP index, a secondary marker of insulin resistance^9^. In this study, when dividing the group of women with PCOS according to levels of hair cortisol, we observed that only women with levels above the reference value showed correlation between androgens and metabolic and inflammatory parameters, as well as between levels of vitamin D with leptin and the TG/HDL index. The hyperactivation of the HPA axis observed in these women, evidenced by hair cortisol measurement, could play a role in this disease pathophysiology.

There are many studies that evaluate hair cortisol as a useful tool in the assessment of Cushing’s syndrome^31^, cyclic Cushing’s syndrome^32^, depression^33^, maternal stress during pregnanacy^34^, as well as in the follow-up of patients with adrenal insufciency treated with hydrocortisone^35^. More recently, it has been shown that hair cortisol results an interesting biomarker of stress and burnout in a health workers population^23^. However, to our knowledge, there are no publications considering hair cortisol levels as a biomarker of HPA hyperactivation in PCOS, although different authors have shown that stress is frequently found in women with this condition^36,37^. Nowadays stress reduction programs have been developed in order to help people to ameliorate this adverse condition. In this sense, in a previous study, our group has shown that hair cortisol concentration results a good biomarker for the evaluation of these programs’ effectiveness^22^.

One limitation of this study is the small number of patients, although it is important to remark that we used strict inclusion criteria. However, we consider this work as an interesting and novel contribution to the knowledge about the rol of hair cortisol in assessing HPA axis hyperactivation in PCOS women.

The results obtained in the current study merit further investigation and the evaluation of patient’s perinatal history in order to relate whether the occurrence of adverse conditions during fetal stage or delivery are related to HPA axis hyperactivation in adult life.

## Conclusion

Hair cortisol measurement is a valuable tool reflecting HPA axis activation in women with PCOS. High hair cortisol levels might be related to hyperandrogenism and metabolic alterations.

## Data Availability

The datasets generated during and/or analyzed during the current study are available from the corresponding author on reasonable request.
